# Multidomain trials to prevent dementia: addressing methodological challenges

**DOI:** 10.1186/s13195-022-01036-1

**Published:** 2022-07-11

**Authors:** Manuel Montero-Odasso, G Y Zou, Nellie Kamkar, Howard H. Feldman, Sylvie Belleville, Howard Chertkow, Haakon B Nygaard, Surim Son, Mark Speechley

**Affiliations:** 1 Schulich School of Medicine and Dentistry, Department of Medicine and Division of Geriatric Medicine, The University of Western Ontario, London, ON Canada; 2 Gait and Brain Lab, Parkwood Institute, Lawson Health Research Institute, London, ON Canada; 3 Department of Epidemiology and Biostatistics, The University of Western Ontario, London, ON Canada; 4 Department of Neurosciences, Alzheimer Disease Cooperative Study, University of California, San Diego, CA USA; 5 Deparment of Psychology, Université de Montréal, Montréal, Québec Canada; 6 Rotman Research Institute, Baycrest Health Sciences, Toronto, ON Canada; 7 Division of Neurology, Charles E. Fipke Integrated Neuroimaging Suite, University of British Columbia, Vancouver, BC Canada

**Keywords:** Multifactorial interventions, Multidomain trials, Combined interventions, Dementia

## Abstract

**Background:**

Multidomain trials to prevent dementia by simultaneously targeting multiple risk factors with non-pharmacological lifestyle interventions show promise. Designing trials to evaluate the efficacy of individual interventions and their combinations is methodologically challenging. Determining the efficacy is, nevertheless, important to individuals, payers, and for resource allocations to support intervention implementation.

**Main body:**

The central rationale for seminal trials improving cardiovascular health or reducing falls risk in older adults is that multifactorial conditions may be amenable to improvement by simultaneously targeting multiple modifiable risk factors. Similar reasoning underlies lifestyle interventions to reduce dementia risk using combinations of physical exercise, cognitive training, diet, amelioration of vascular-metabolic risk factors, and improving sleep quality. Randomizing individuals with at least two modifiable risk factors to “standardly tailored” interventions to mitigate their risk factors, versus a comparator arm, will yield an unbiased estimate of the cumulative average effect of modifying more versus fewer risk factors. The between-group difference in the cognitive primary outcome will reflect both the main effects of the mitigated risk factors, as well as their synergistic effects. However, given the positive trial results, there are inherent challenges in quantifying post hoc which components, or combination of components, were responsible for improvements in cognition. Here, we elaborate on these methodological challenges and important considerations in using a standardly tailored design with two arms (one consisting of multidomain interventions tailored to participants’ risk profiles and another consisting of active control conditions). We compare this approach to fully factorial designs and highlight the disadvantages and advantages of each. We discuss partial solutions, including analytical strategies such as risk reduction scores that measure reductions in the number or severity of risk factors in each study arm. Positive results can support the causal inference that between-group differences in the primary cognitive outcome were due to risk factor modification.

**Conclusion:**

Standardly tailored designs are pragmatic and feasible evaluations of multidomain interventions to reduce dementia risk. We propose sensitivity and exploratory analyses of between-group reductions in the severity of risk factors, as a methodology to bolster causal inferences that between-group differences in the primary cognitive outcome are due to the risk factors modified.

**Supplementary Information:**

The online version contains supplementary material available at 10.1186/s13195-022-01036-1.

## Background

Multidomain trials of non-pharmacological interventions targeting multiple risk factors in the cognitive decline of aging are showing considerable promise [1]. Emerging research suggests that multiple lifestyle changes, including physical activity, managing vascular risk factors, and engaging in cognitive training activities, may improve cognition and potentially delay, if not prevent, dementia’s onset [1–4]. Additional protective factors include maintaining a brain healthy diet and regularly attained sleep [1–5]. Designing trials to evaluate multidomain interventions is more methodologically challenging than typical single intervention randomized controlled trials (RCTs). Plausible interpretations of positive multidomain trials are also more complicated.

## Main text

Multidomain trials targeting treatable lifestyle risk factors for dementia in the community include the seminal FINGER trial (Finnish Geriatric Intervention Study to Prevent Cognitive Impairment and Disability) in which participants were randomized to a “basket” of interventions including physical exercises, cognitive training, vascular risk factor control, and nutritional counseling, compared to a control group receiving general health advice [1]. This approach reflects both the probable multifactorial causality of dementia and the potential for combined interventions to operate synergistically.

Previous clinical trials targeting multiple risk factors for conditions such as heart disease and falls in older adults often used the “standardly tailored” design that involves randomizing participants with two or more modifiable risk factors to receive interventions targeted at their risk factors, or usual care [5]. Differences in the primary outcome between randomized groups are an unbiased estimate of the cumulative average effect of reducing more risk factors in one group than in the other. Risk factors tend to cluster in individuals, and synergistic causal interactions occur among specified combinations of risk factors, as seen with heart disease and falls [6, 7].

The “standardly tailored” design will produce positive results if the interventions reduce the frequency and/or severity of the targeted risk factors, and the risk factors are causal. When a main effect is reduced, synergistic effects with other factors are reduced as well. Thus, the “standardly tailored” design yields an unbiased estimate of the net effect (main effects and interactions) of modifying risk factors, as compared to not modifying them. It cannot, however, produce unbiased effect estimates for any of the dozens of possible combinations of effectively modified risk factors.

**Fig. 1:**
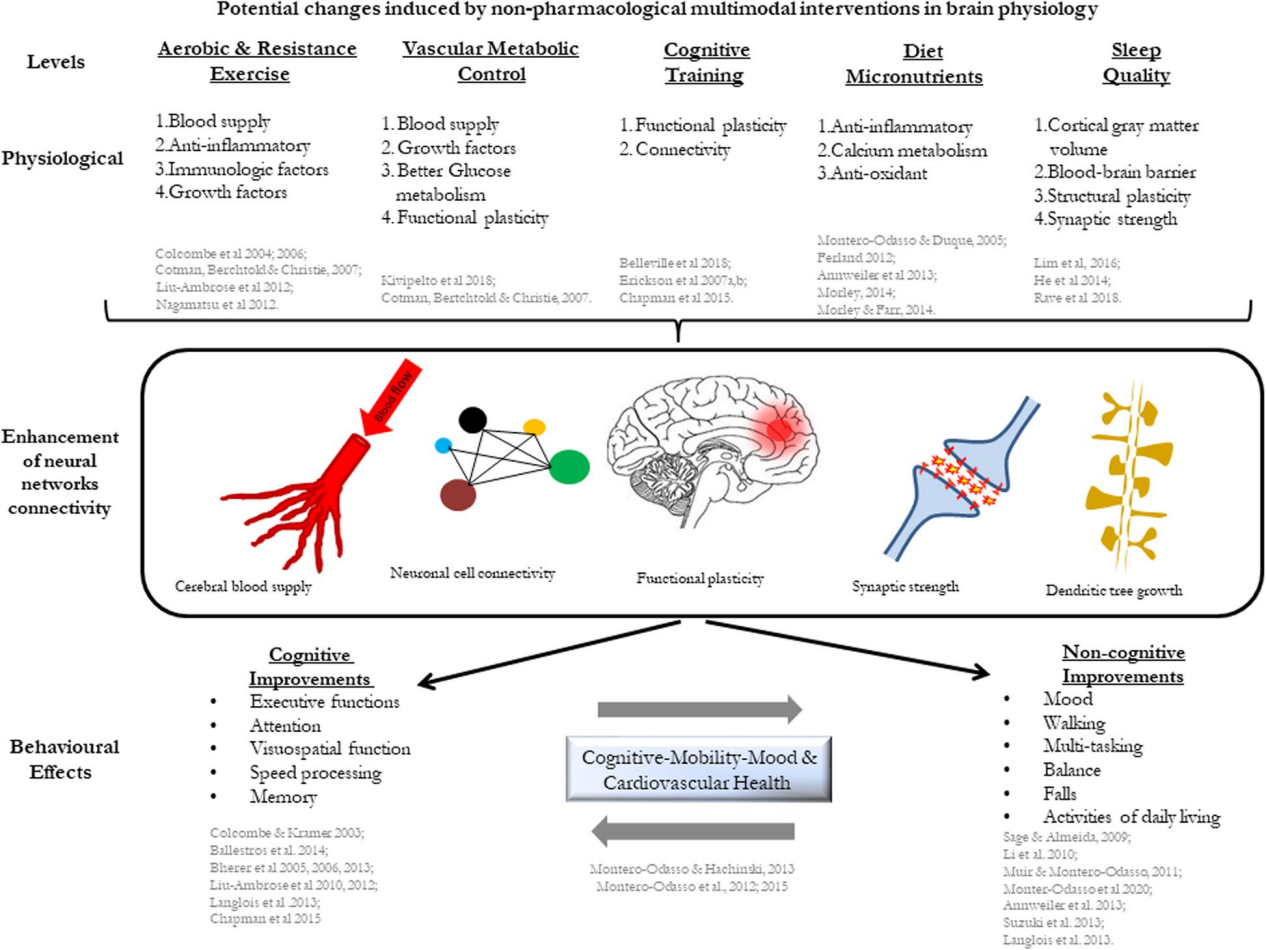
Potential physiological and brain changes following multidomain interventions in dementia prevention

These lifestyle interventions that target multiple pathological mechanisms may be better suited for primary and secondary dementia prevention. This is the case for tailoring physical exercise, cardiovascular and metabolic control, diet and sleep, and cognitive training that have been shown to improve brain microvascularity, neuroplasticity, and neuroconnectivity, with reduction of neural inflammation (Fig. 1) [8].

### Limitations and a solution

Nevertheless, the “standardly tailored” design presents some challenges. In the event of successful multiple intervention clinical trials in chronic diseases (e.g., coronary artery disease, falls), researchers and policymakers will wish to know the effect sizes of both individual and combinations of modified risk factors. The “standardly tailored” design cannot yield unbiased estimates of the effects of modifying any one risk factor alone or any combination of risk factors, because strata formed by these risk factor subgroups have not been the basis for an independent, random assignment to intervention and control conditions. Conversely, the optimal design for unbiased estimates of both main effects and interactions is the fully factorial randomized controlled trial, which has two major limitations in such studies.

One limitation is a sample size. A study with the five potentially modifiable lifestyle factors, such as detailed above for dementia prevention, would require 32 separated arms (2^5^) to estimate the main effect of each intervention and all possible interactions. Having 32 arms is impractical due to the large sample size required to achieve acceptable statistical power between arms. In addition, it is inefficient because it expends degrees of freedom estimating many higher-order interactions of no scientific interest or practical clinical applicability.

A second limitation is that a full factorial trial of five factors would require participants to be “at risk” for all five factors (i.e., sedentarism, at least one vascular factor, such as hypertension, an unhealthy diet, poor sleep, and lack of cognitively challenging activities) so that each subgroup can be independently randomized. This substantially limits generalizability of the results, because most people will not be at risk for all five factors, and this requirement would severely complicate recruitment even from a trial-ready cohort [9, 10].

**Fig. 2:**
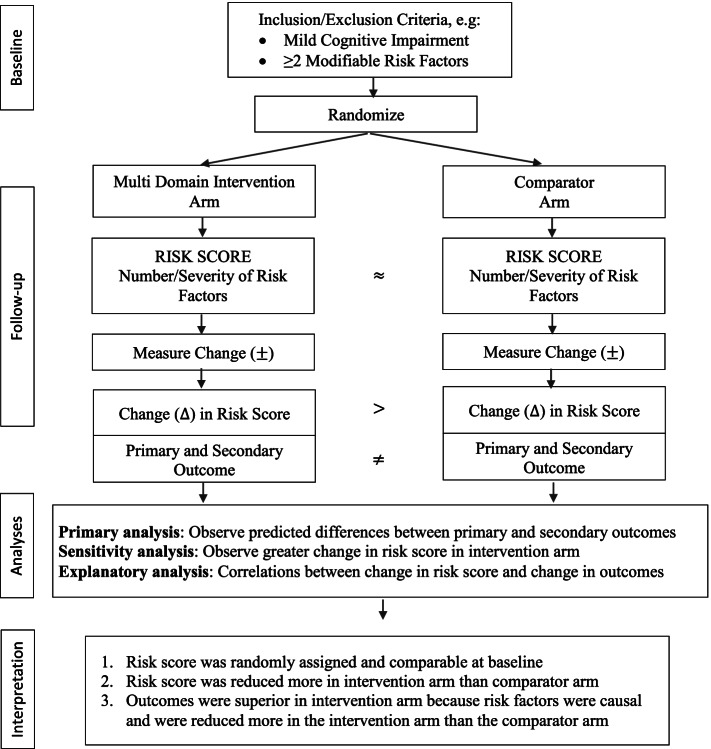
Representation of how “risk reduction scores” can be included in the analyses and interpretation of results when designing “standardly tailored’” trials targeting multiple risk factors, as we have designed for the SYNERGIC-2 Trial (CT.gov #3948). MCI, mild cognitive impairment

One possible solution to this challenge is by using a *risk reduction score* for each domain to be targeted, as has been demonstrated in previous multidomain trials in fall prevention [11, 12]. These *risk reduction scores* can be produced by subtracting the post-intervention performance score in the given domain from the baseline performance, reflecting the changes seen in each domain as a “delta” (Δ) change. Figure 2 shows an example of how *risk reduction scores* can be applied in the analyses and interpretation of results when designing the “standardly tailored” trial targeting multiple risk factors, as we have designed for the SYNERGIC-2 Trial (CT.gov #3948). The *risk reduction score* will allow examination of the correlations between changes in risk for each domain and the effect of the changes on the primary and secondary outcomes selected. It would also allow evaluation of potential mediators of the treatment effect, by using multivariable models that include baseline risk scores and other important covariates. Of course, covariates need to be pre-specified to avoid bias caused by convential model selection procedures commonly seen in epidemiological studies. Because some interventions to prevent dementia, like exercise, may have plausible synergistic effects with other interventions, fitting the other intervention terms in the model with the intervention can address plausible synergisms. In addition, timing of the interventions should also be considered because the importance of various risk factors may vary across the life-cycle, e.g., midlife, older at-risk individuals, persons with early Alzheimer’s disease. Similarly, the intensity and dose of the intervention for individual components of the interventions needs to be measured since a “dose–response function” has been recently described in multidomain interventional trials aimed to improve cognition. For instance, analyses of the MAPT trial showed that the optimal dose in training for exercises, cognitive, and nutrition intervention was half of the potentially available dose, and attainment of a plateau in primary outcome change was found after that optimal dose was reached [13].

## Conclusions

While there are disadvantages associated with multidomain interventions, including the common argument that it is not possible to determine which component or components were responsible for the treatment effect, standardly tailored trials simultaneously targeting multiple risk factors are appealing because most risk factors for dementia are chronic that may be amenable to amelioration, but not elimination. Although it is not possible to estimate the effect of the individual component of the combined interventions, using risk reduction scores or change as a function of intensity and dose can help to estimate the effect in each domain targeted and may allow researchers to address mediation effects. Tailoring interventions to each individual’s combination of risk impairments may provide a more realistic and effective primary and secondary prevention interventional strategy, aligned with the “precision medicine” paradigm [14].

## Supplementary Information

**Additional file 1:** **Supplementary Table 1.** References in Figure 1.

## Data Availability

Not applicable (no dataset analyzed).

## Abbreviations

FINGER Trial: Finnish Geriatric Intervention Study to Prevent Cognitive Impairment and Disability
MCI: Mild cognitive impairment
