# Supplementary material for: Multidomain trials to prevent dementia: addressing methodological challenges
Source: Alzheimers Res Ther. 2022 Jul 11;14:94. doi: 10.1186/s13195-022-01036-1 (PMC9275261; doi:10.1186/s13195-022-01036-1)
Supplement: Supplementary file 1 — Additional file 1: Supplementary Table 1. References in Figure 1. [file 13195_2022_1036_MOESM1_ESM.docx]

**SUPPLEMENTARY MATERIALS**

**Supplementary Table 1.** References in Figure 1.

| **Subsection in Figure 1** | **Reference Citation** |
| --- | --- |
| **Aerobic and Resistance Exercise** | Colcombe et al., 2004 |
|  | Colcombe et al., 2006 |
|  | Liu-Ambrose et al., 2012 |
|  | Nagamatsu et al., 2012 |
| **Vascular and Metabolic Control** | Kivipelto et al., 2018 |
|  | Cotman, Bertchtold & Christie, 2007 |
| **Cognitive Training** | Belleville et al., 2018 |
|  | Erickson et al., 2007a |
|  | Erickson et al., 2007b |
|  | Chapman et al., 2015 |
| **Diet Micronutrients** | Montero-Odasso & Duque, 2005 |
|  | Ferland, 2012 |
|  | Annweiler et al 2013 |
|  | Morley et al., 2014 |
|  | Morley & Farr, 2014 |
| **Sleep Quality** | Lim et al., 2016 |
|  | He et al., 2014 |
|  | Rave et al., 2018 |
| **Cognitive Improvements** | Colcombe & Kramer 2003 |
|  | Ballestros et al. 2014 |
|  | Bherer et al., 2005 |
|  | Bherer et al., 2006 |
|  | Bherer et al., 2013 |
|  | Liu-Ambrose et al., 2010 |
|  | Liu-Ambrose et al., 2012 |
|  | Langlois et al., 2013 |
|  | Chapman et al., 2015 |
| **Cognitive-Mobility-Mood & Cardiovascular Health** | Montero-Odasso & Hachinski, 2013 |
|  | Montero-Odasso et al., 2012 |
|  | Montero-Odasso et al., 2015 |
| **Non-Cognitive Improvements** | Sage & Almeida, 2009 |
|  | Li et al., 2010 |
|  | Muir & Montero-Odasso, 2011 |
|  | Montero-Odasso et al., 2020 |
|  | Annweiler et al. 2013 |
|  | Suzuki et al., 2013 |
|  | Langlois et al., 2013 |
